# Supplementary material for: SLE non-coding genetic risk variant determines the epigenetic dysfunction of an immune cell specific enhancer that controls disease-critical microRNA expression
Source: Nat Commun. 2021 Jan 8;12:135. doi: 10.1038/s41467-020-20460-1 (PMC7794586; doi:10.1038/s41467-020-20460-1)
Supplement: Supplementary file 1 — Supplementary information [file 41467_2020_20460_MOESM1_ESM.pdf]

Hou *et al.*

SLE non-coding Genetic Risk Variant Determines the Epigenetic Dysfunction of an Immune Cell Specific Enhancer that Controls Disease-critical microRNA Expression

Supplementary information

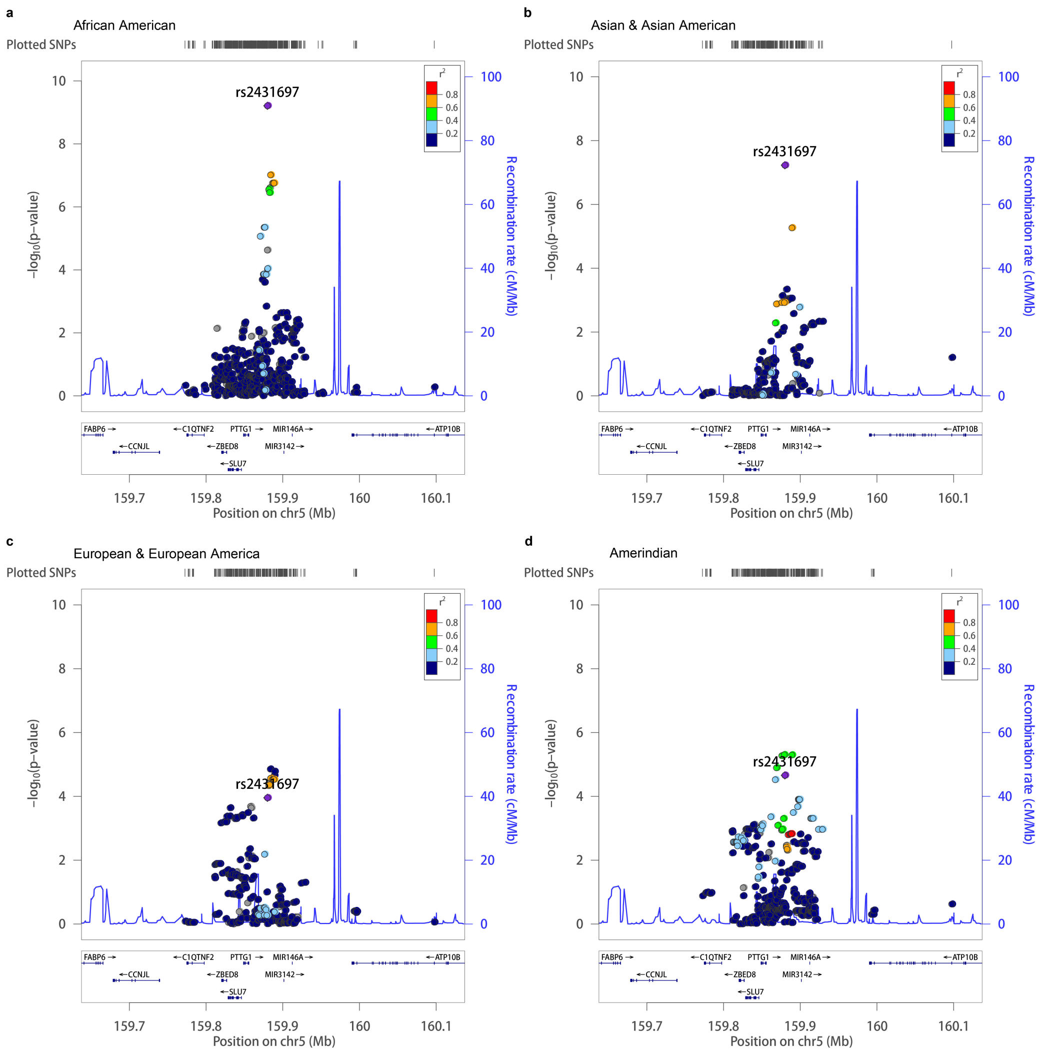

Supplementary Fig. 1 | Related to Fig. 1, Intergenic variants between *PTTG1* and *MIR3142HG* demonstrate genome-wide association with SLE in a multi-ethnic discovery cohort. Each data point represents a variant in its genomic position (GRCh37/hg19) and the strength of association with SLE. Strength of association is assessed as -log10 *P*-value in logistic regression models using admixture estimates as a covariate. Data point coloring is on the basis of linkage disequilibrium with the most strongly associated variant in the trans-ancestral meta-analysis, 5:159879978:T:C (dbSNP refSNP id: rs2431697) in the respective ancestral population (a – African American Ancestry, b – Asian & Asian American Ancestry, c – European & European American Ancestry, d – Amerindian Ancestry) from the hg19/1000 Genomes 2014 data available on http://locuszoom.org as indicated in the legend. Genome-wide association is defined here as *P* < 5E10-8.

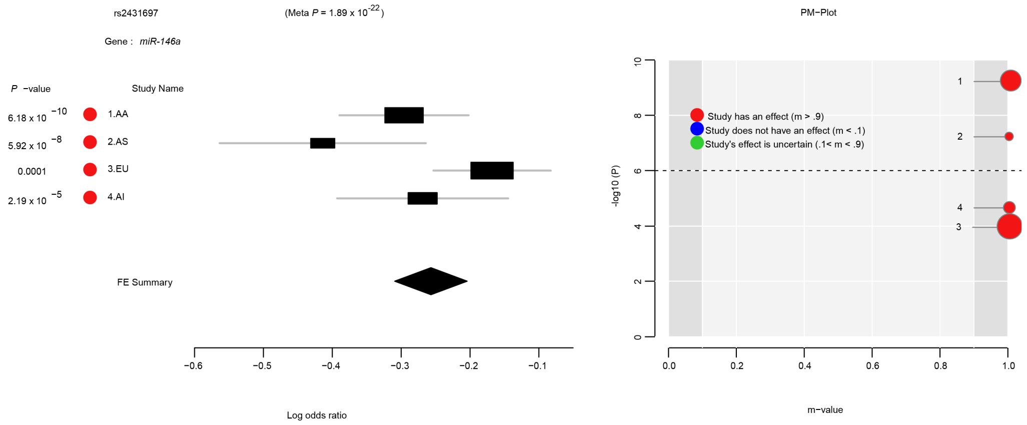

Supplementary Fig. 2 | Related to Fig. 1, Forest Plot and PM-Plot of rs2431697 in Discovery Cohort using fixed effects meta-analysis based on inverse variance weighted effect size, as implemented in METASOFT[1,2] and ForestPMPlot[3]. Since the test statistic (Z_{FE}) follows the standard normal distribution under the null hypothesis of no association, this test is a two-tailed test[3]. *P*-values presented are not adjusted for multiple comparisons. AA = African American, AS = Asian & Asian American, EU = European & European American, AI = Amerindian.

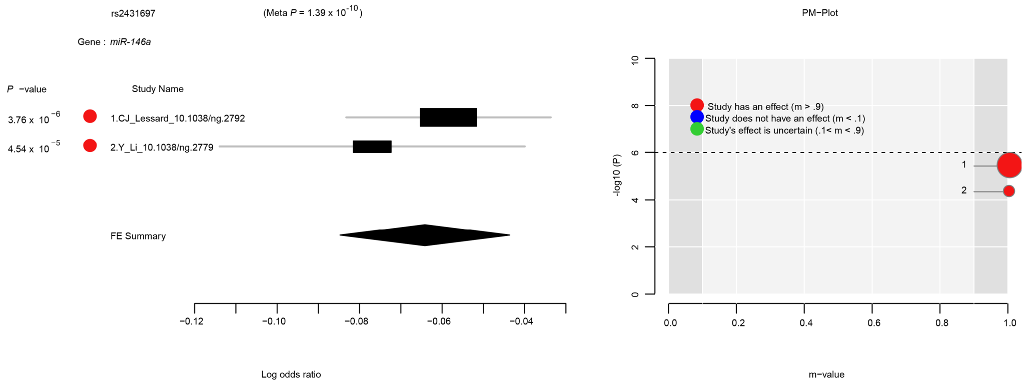

Supplementary Fig. 3 | Related to Fig. 1, Meta-analysis of two independent GWAS of Sjogren's Syndrome define rs2431697-G as a protective allele. Meta-analysis as according to fixed effects meta-analysis based on inverse variance weighted effect size, implemented in METASOFT and ForestPMPlot. Since the test statistic (Z_{FE}) follows the standard normal distribution under the null hypothesis of no association, this test is a two-tailed test[3]. Studies include: CJ_Lessard_10.1038/ng.2792[4] and Y_Li_10.1038/ng.2779[5]. *P*-values presented are not adjusted for multiple comparisons.

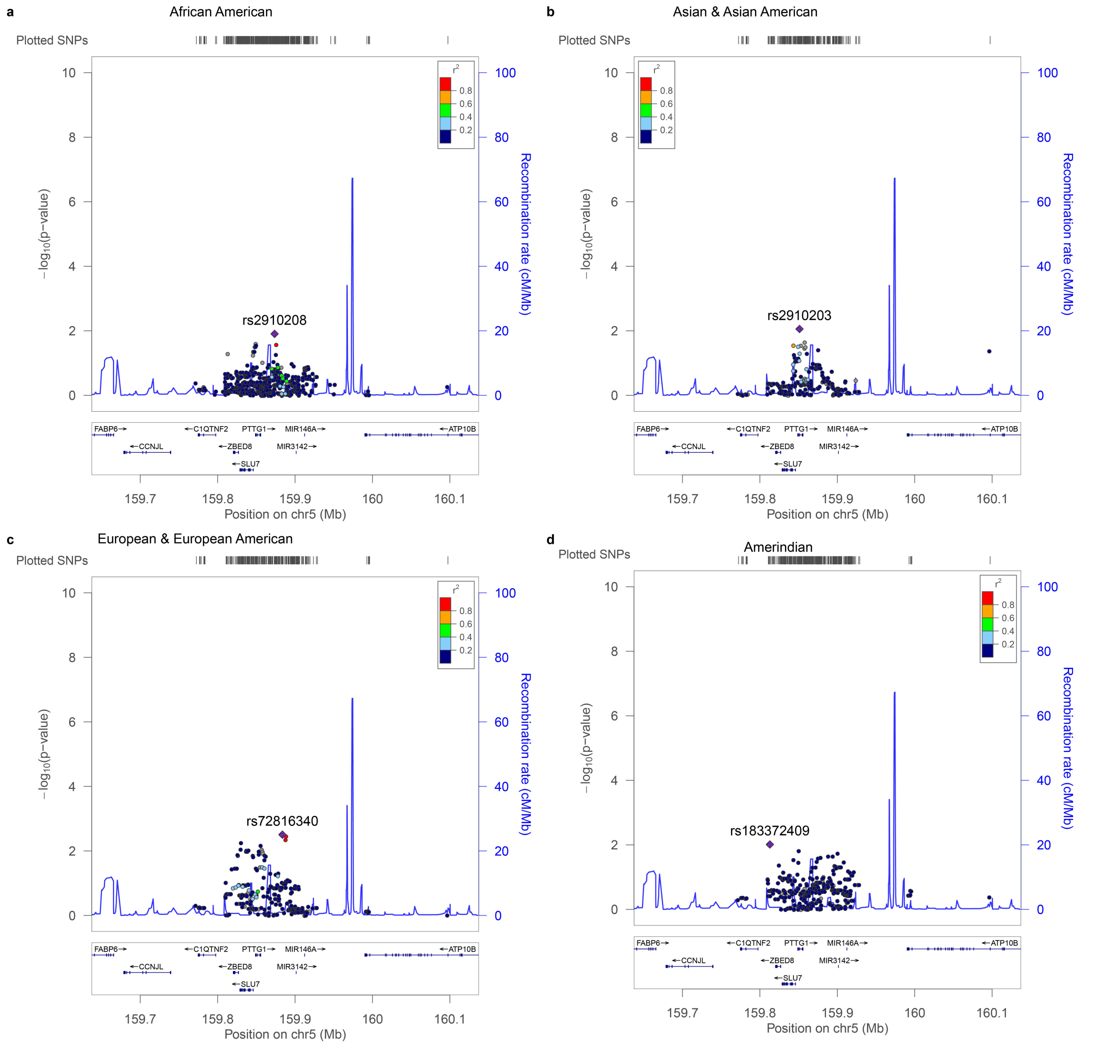

Supplementary Fig. 4 | Related to Fig. 1, Conditional logistic regression reveals minimal residual association signal beyond that tagged by rs2431697, likely complex in nature. Logistic regression analysis, including admixture estimates and rs2431697 as covariates in the model. Analysis performed in each of the indicated populations. Data points represent variants and coloring is on the basis of linkage disequilibrium with the most strongly associated variant after conditioning on the genotype of rs2431697 in the respective ancestral population (a – African American Ancestry, b – Asian & Asian American Ancestry, c – European & European American Ancestry, d – Amerindian Ancestry) from the hg19/1000 Genomes 2014 data available on http://locuszoom.org as indicated in the legend.

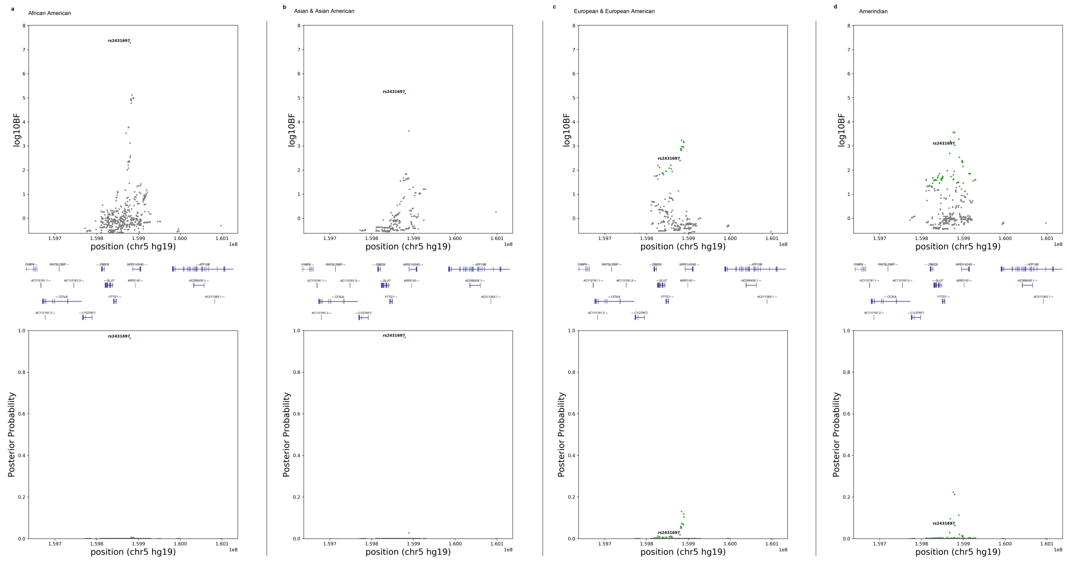

Supplementary Fig. 5 | Related to Fig. 1, Discovery cohort Bayesian analysis identifies a single genetic variant, rs2431697, that is present in the 95% credible set in all ancestral groups analyzed. Each data point represents a variant in its genomic position (GRCh37/hg19) and the strength of association with SLE. Strength of association is assessed as the posterior probability that the variant (or an untyped, unimputed markers) is causal (**a-d**, top panel) and the $\log 10$ Bayes Factor using admixture estimates as a covariate. (**a-d**, bottom panel). Variants in green represent members of the 95% credible set for each ancestry. The remainder of variants not present in the 95% credible set are in grey. Chr, chromosome. (**a**) African American Ancestry, (**b**) Asian & Asian American Ancestry, (**c**) European & European American Ancestry, (**d**) Amerindian Ancestry.

a African American

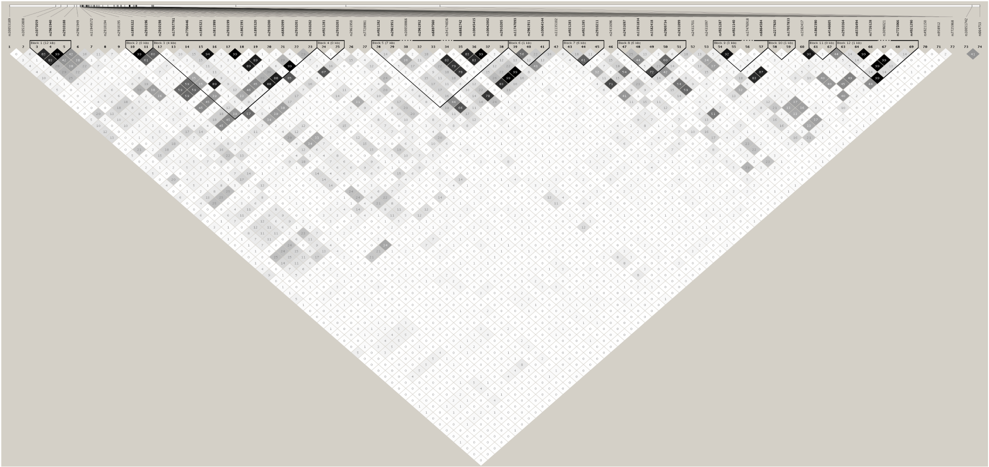

b Asian & Asian American

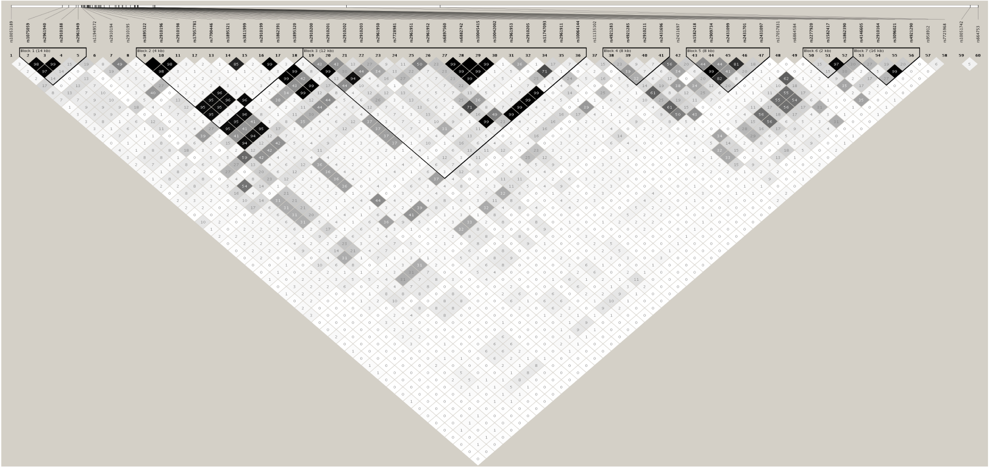

c European & European American

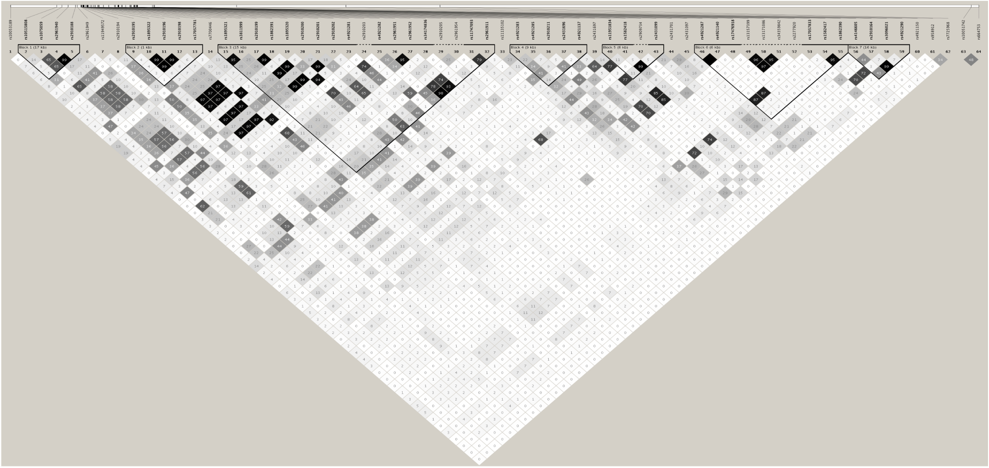

d Amerindian

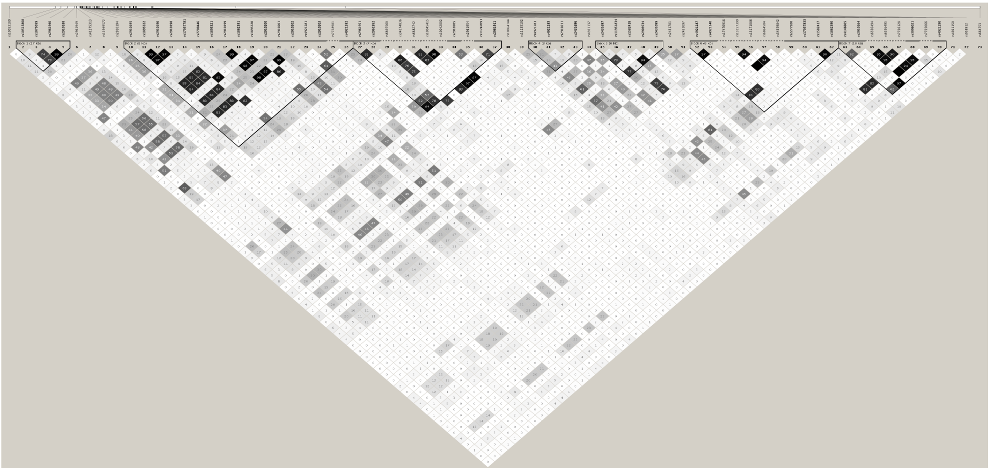

Supplementary Fig. 6 | Related to Fig 1, LD structure in 4 ancestral population case-control collections from the discovery cohort (LLAS2 study). (**a-d**) LD of genotyped variants within ancestral population indicated in figure. These figures were generated using genotype data combining cases and controls by ancestry with Haploview (v4.1). Shading is according to the degree of linkage disequilibrium (LD) with the r^2 color scheme (darker shading indicates stronger LD, that is r^2 closer to 1).

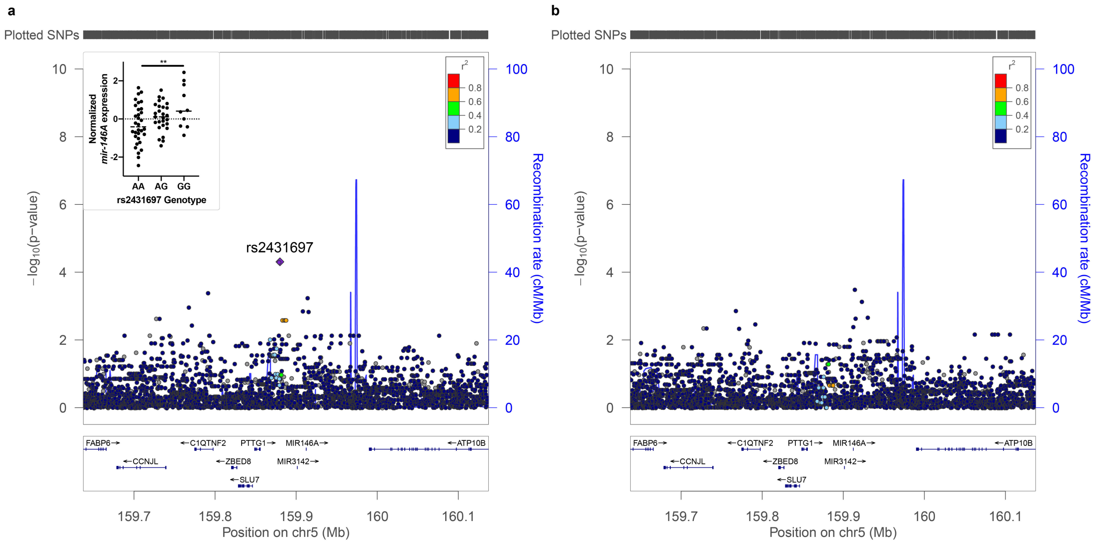

Supplementary Fig. 7 | Related to Fig 1, eQTL analysis of ENSG00000207936/miR-146a with expression values from Pickrell JK, et al., re-analyzed with 1000 genomes project genotype data. a, Unadjusted Linear regression analysis relating genotype to normalized, PC-corrected miR-146a expression values of LCL cells. **b,** Linear regression analysis conditioning on rs2431697 genotype in the linear model. Data points represent a single variant and coloring is on the basis of linkage disequilibrium with rs2431697 from the AFR (African American) population from hg19/1000 Genomes 2014 data available on http://locuszoom.org as indicated in the legend. Expression data are from Pickrell JK, Nature 2010[6], re-analyzed with 1000 genomes project genotype data on subset of 58 individuals of Yoruban ancestry from Ibadan, Nigeria which overlapped between these two data sets. Inset: Normalized, PC-corrected miR-146a expression values plotted by genotype ** $P < 0.01$, One-Way ANOVA test for linear trend.

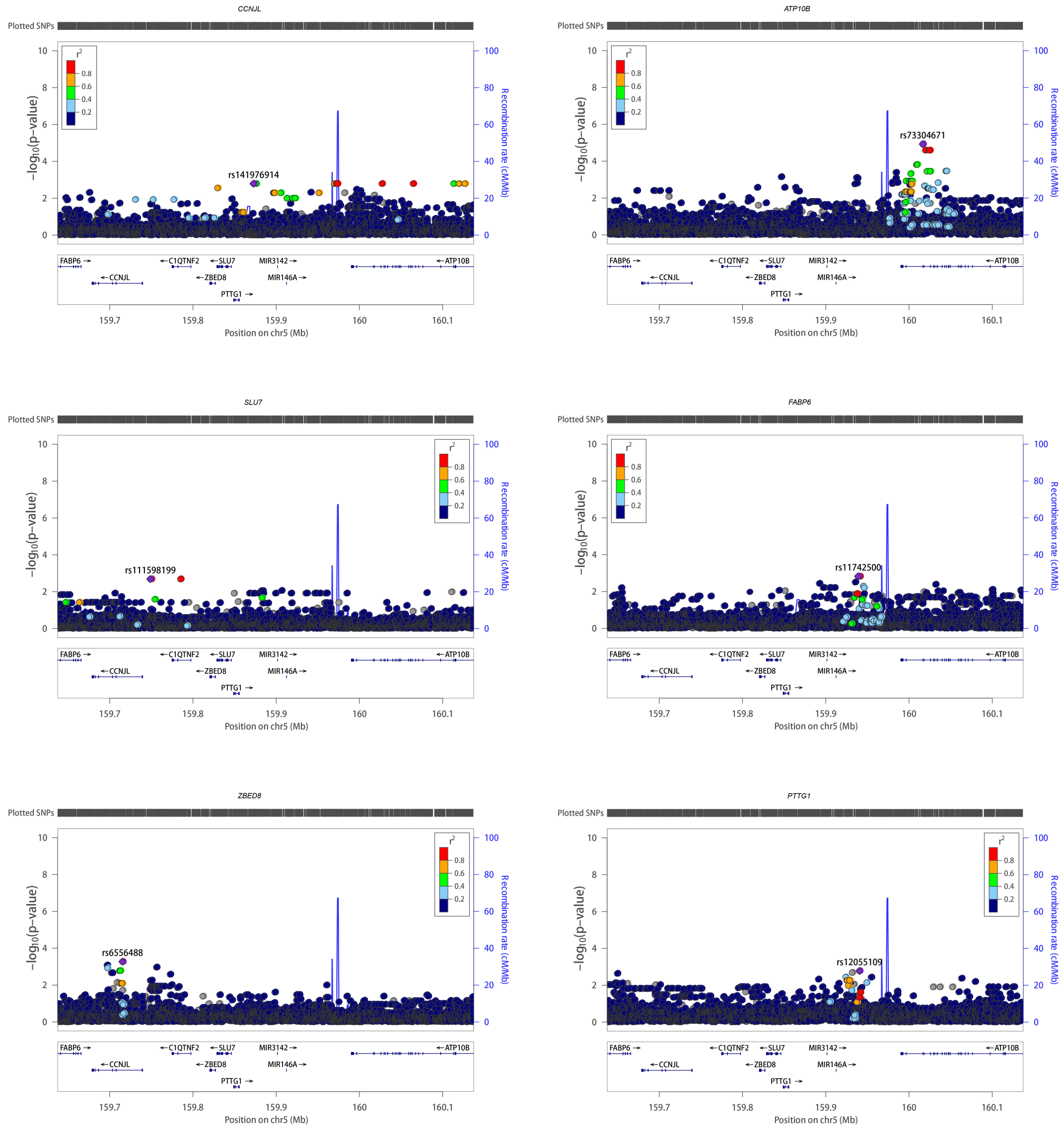

Supplementary Fig. 8 | Related to Fig 1, Linear regression analysis reveals that SLE risk locus does not overlap with nominal eQTLs of other genes in the *PTTG1-MIR3142HG* region in lymphoblastoid cell lines. Unadjusted Linear regression analysis relating genotype to normalized, PC-corrected miR-146a expression values of LCL cells for other genes in the region. Titles indicate HGNC (HUGO Gene Nomenclature Committee) gene name and blue highlighting emphasizes the transcript used as the response variable in the linear model relating genotype to gene expression in these cells. Expression data are from Pickrell JK, Nature 2010[6], re-analyzed with 1000 genomes project genotype data on subset of 58 individuals of Yoruban ancestry from Ibadan, Nigeria which overlapped between these two data sets. Each data point represents a variant in its genomic position (GRCh37/hg19) and the strength of association with expression of the indicated gene. Strength of association is assessed as -log10 *P*-value in the linear regression model. The data points are colored on the basis of linkage disequilibrium with the most strongly associated variant in the AFR (African American) ancestral population from the hg19/1000 Genomes data available on http://locuszoom.org. Note: *C1QTNF2* did not

appear to have been mapped in this RNA-Seq data set, suggesting that it was not robustly expressed in these cell lines.

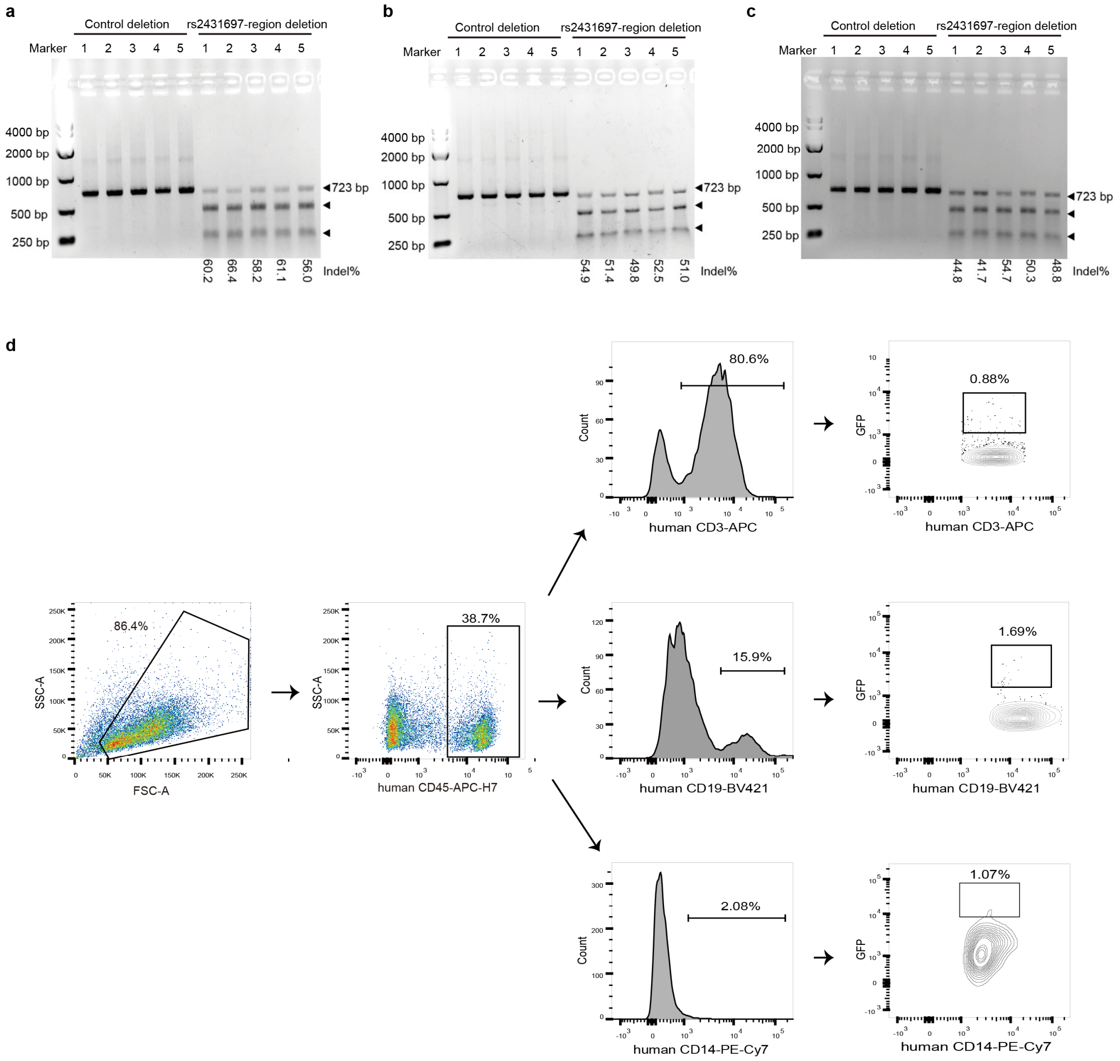

Supplementary Fig. 9 | Related to Fig. 3, a-c, T7 endonuclease I (T7E1) assay demonstrates genome editing efficiency in the rs2431697-containing region in CD3+ T cells (**a**), CD19+ B cells (**b**) and CD14+ monocytes (**c**). Expected PCR product size (723 nt) and approximate expected sizes of T7E1-digested fragments are indicated. **d,** Representative FACS for sorting GFP+ CD14+ monocytes, GFP+CD19+ B cells and GFP+CD3+ T cells. GFP+ indicates cells transfected with the Adenovirus Cas9-GFP-sgRNA.

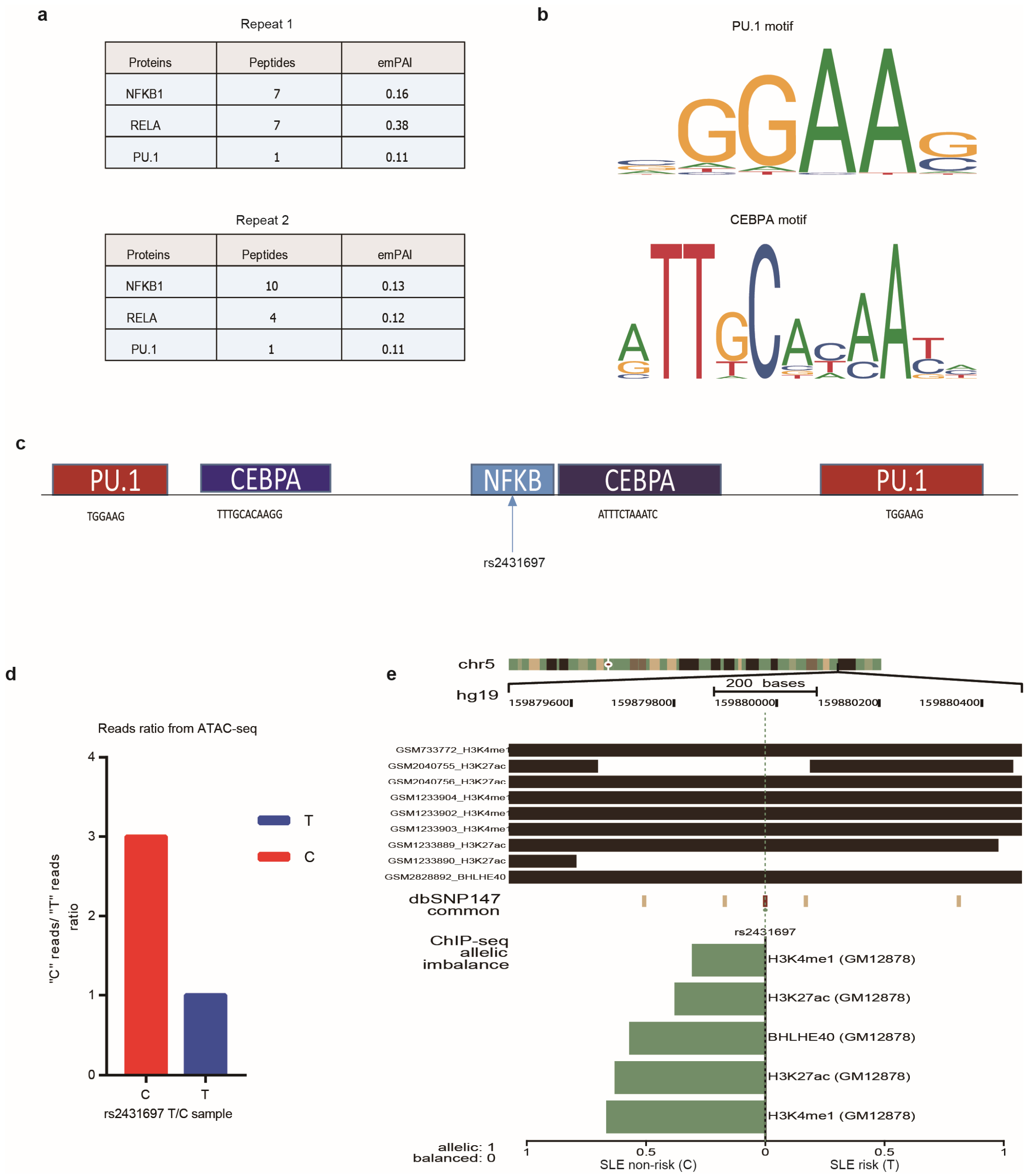

Supplementary Fig. 10 | Related to Fig. 5, a, MS identifies the major transcription factors enriched by 41-bp DNA sequence harboring rs2431697 C allele of two replicate results (emPAI, Exponentially Modified Protein Abundance Index). **b,** DNA binding motif of PU.1 and CEBPA analyzed by JASPAR. **c,** Possible binding transcription factors nearby rs2431697 analyzed by bioinformatics analysis. **d,** Fragments harboring C allele has more reads than fragments harboring T allele detected by ATAC-seq. **e,** Allelic distribution of H3K4me1 and H3K27ac at rs2431697 site. Chr, chromosome.

Supplementary Table 1

sgRNA sequences and HDR template for CRISPR assay

Experiment	sgRNA description	Sequence 5' to 3'
Fragment harboring rs2431697 deletion	sgRNA1	GAACTCAGTGCACATGACAT
Fragment harboring rs2431697 deletion	sgRNA2	TGTATCAGATTTCTAAATCG
distal promotor deletion	sgPRo1L	GTCCCTTCTAGATCCCTCCT
distal promotor deletion	sgPRo1R	GAGGGTTAGCGTGCAGGGTG
proximal promoter deletion	sgPRo2L	GTTTATAACTCATGAGTGCC
proximal promoter deletion	sgPRo2R	GTACTAGGAAGCAGCTGCAT
rs2431697 HDR	sgHR	TTGTATCAGATTTCTAAATC
CRISPRa assay	sg1a	TTCATATGGATATTTGCACA
CRISPRa assay	sg2a	ATATACGTGTATGACAAAGT
CRISPRi assay	sg1i	AAAACTTGGCTCTGCTTCAT
CRISPRi assay	sg2i	CTTGCCTCGTGCTTCTGCAC
CRISPR assay	sgNC1	GTTCCGCGTTACATAACTTA
CRISPR assay	sgNC2	ACTACAAGTAAAAGTATCGG

rs2431697 HDR template

AGAGGGGGTGAAAGAAGGAACTCAGTGCACATGACATTGGTGGGGCTGAAATAAAAAACCTCGATTTAGAAATC
TGATACAAAAGCAAAGTCATCGTTTTCAAATCAAAACTTGGCTCTGC

red T is rs2431697

Supplementary Table 2

Primer sequences used in this paper

Experiment	primer description	Sequence 5' to 3'
RT-qPCR	hsa-pri-miR-146a-qpcr-F	TGAGAACTGAATTCCATGGGTT
RT-qPCR	hsa-pri-miR-146a-qpcr-R	ATCTACTCTCTCCAGGTCCTCA
RT-qPCR	hsa-IFIT1-qpcr-F	TTGATGACGATGAAATGCCTGA
RT-qPCR	hsa-IFIT1-qpcr-R	CAGGTCACCAGACTCCTCAC
RT-qPCR	hsa-IFIT3-qpcr-F	TGAGGTCACCAAGAATTCCCTG
RT-qPCR	hsa-IFIT3-qpcr-R	CAATCTGGTTACACACTCTATCTTC
RT-qPCR	hsa-OAS1-qpcr-F	TGTCCAAGGTGGTAAAGGGTG
RT-qPCR	hsa-OAS1-qpcr-R	CCGGCGATTTAACTGATCCTG
RT-qPCR	hsa-IFI44-qpcr-F	ATGGCAGTGACAACTCGTTTG
RT-qPCR	hsa-IFI44-qpcr-R	TCCTGGTAACTCTCTTCTGCATA
RT-qPCR	hsa-PWWP2A-qpcr-F	CTTGTCGTGTCGTTCCGCTT
RT-qPCR	hsa-PWWP2A-qpcr-R	ACCATTTGCTTCACACTTGACTT
RT-qPCR	hsa-TTC1-qpcr-F	GAGCGGACAAGGTTGAGAACA
RT-qPCR	hsa-TTC1-qpcr-R	CTCCTCCTTTAGTCTAGTGCTCT
RT-qPCR	hsa-SLU7-qpcr-F	AAGAACAGCGAAAATTGGGCA
RT-qPCR	hsa-SLU7-qpcr-R	CCCTCTTGTACCATTCTCCAGA
RT-qPCR	hsa-UBLCP1-qpcr-F	CTCGCAGAGTGAAAGAGTACAAA

RT-qPCR	hsa-UBLCP1-qpcr-R	GCACAAGACCTGTGGTCAAATA
RT-qPCR	hsa-GAPDH-qpcr-F	GGAGCGAGATCCCTCCAAAAT
RT-qPCR	hsa-GAPDH-qpcr-R	GGCTGTTGTCATACTTCTCATGG
RT-qPCR	hsa-PTTG1-qpcr-F	ACCCGTGTGGTTGCTAAGG
RT-qPCR	hsa-PTTG1-qpcr-R	ACGTGGTGTTGAAACTTGAGAT
RT-qPCR	hsa-ly6e-qpcr-F	CAGCTCGCTGATGTGCTTCT
RT-qPCR	hsa-ly6e-qpcr-R	CAGACACAGTCACGCAGTAGT
RT-qPCR	hsa-IFI27-qpcr-F	TGCTCTCACCTCATCAGCAGT
RT-qPCR	hsa-IFI27-qpcr-R	CACAACTCCTCCAATCACAACT
Genotype	distal promoter-F	CAGCGCCTGACCAGAACTTC
Genotype	distal promoter-R	TCCATCCTGTCCACCCTTTG
Genotype	proximal promoter-F	CTGAGGAAGTGACATTGAAAGCA
Genotype	proximal promoter-R	CAAGCCCACGATGACAGAGATA
Genotype	697-genotype-F	GGTTTTAAGAACTGAAACTTGGGA
Genotype	697-genotype-R	AGGCAAGCCAATGAAGCAGA
T7EI assay	697-T7EI-F	GTGTCCTAAGGGTGAGACAAGG
T7EI assay	697-T7EI-R	TAGGAGCACCTACCCCAAGTT
ChIP-qPCR	rs697-chip-F	TGACATTGGTGGGGCTGAAA
ChIP-qPCR	rs697-chip-R	CACGAGGCAAGCCAATGAAG
FAIRE-qPCR	rs697-FAIRE2000-F	CTGCTTCCCTCCAGAGTTGG
FAIRE-qPCR	rs697-FAIRE2000-R	CTAGCCATGCCAGAGACCTG
FAIRE-qPCR	rs697-FAIRE800-F	TCAGGGCTTCTGACAGGTTG
FAIRE-qPCR	rs697-FAIRE800-R	TGGAGGTGGAAATGGGGGTA
FAIRE-qPCR	rs697-FAIRE400-F	CCTCTGTAGCTCCTCCACCT
FAIRE-qPCR	rs697-FAIRE400-R	AGCTGAGCACAGAAACGGAA
FAIRE-qPCR	rs697-FAIRE-F	TGACATTGGTGGGGCTGAAA
FAIRE-qPCR	rs697-FAIRE-R	CACGAGGCAAGCCAATGAAG
FAIRE-qPCR	rs697-FAIRE-500-F	CACTGAGGGTCTTGGGTTCC
FAIRE-qPCR	rs697-FAIRE-500-R	AAGACTTGTGGGCTTGCCTT
FAIRE-qPCR	rs697-FAIRE-900-F	AGCCTGGGCAACATAGTGAG
FAIRE-qPCR	rs697-FAIRE-900-R	AGAAGCAAATGTGGCAAAATG
FAIRE-qPCR	rs697-FAIRE-2000-F	TCTCCTTATTGGAAAGCCGGA
FAIRE-qPCR	rs697-FAIRE-2000-R	AGGCATAAAGCGGCTGAACT
allele-specific qPCR	rs2431697(T)-F	GGGGCTGAAATAAAAAACCT
allele-specific qPCR	rs2431697-common-R	CCAATGAAGCAGAGCCAAGT
allele-specific qPCR	rs2431697(C)-F	GGGGCTGAAATAAAAAACCC
4C-Seq	697-4C-CN-R	GTAGCCTGATCCTAGCTC
4C-Seq	697-4C-CN-F	CTACAGATAAGGTTCTGTAC
4C-Seq	dp-4c-DN-R	CGCTAACCCTCTCTGGAAAGTC
4C-Seq	dp-4c-DN-F	CTTTCTCCAAGACGCTTGAC

Supplementary Table 3

Oligo sequences for EMSA and DAPA

Experiment	Description	Sequence 5' to 3'
EMSA	Rela snpC-F	TGGGGCTGAAATAAAAAACCCCGATTTAGAAATCTGATACA
EMSA	Rela snpC-R	TGTATCAGATTTCTAAATCGGGGTTTTTTATTTCAGCCCCA
EMSA	Rela snpT-F	TGGGGCTGAAATAAAAAACCTCGATTTAGAAATCTGATACA
EMSA	Rela snpT-R	TGTATCAGATTTCTAAATCGAGGTTTTTTATTTCAGCCCCA
DAPA	Rela snpC-F	TGGGGCTGAAATAAAAAACCCCGATTTAGAAATCTGATACA
DAPA	Rela snpC-R	TGTATCAGATTTCTAAATCGGGGTTTTTTATTTCAGCCCCA

Supplementary References

1. Han, B. & Eskin, E. Interpreting meta-analyses of genome-wide association studies. *PLoS Genet* **8**, e1002555 (2012).
2. Han, B. & Eskin, E. Random-effects model aimed at discovering associations in meta-analysis of genome-wide association studies. *Am J Hum Genet* **88**, 586-98 (2011).
3. Kang, E.Y. *et al.* ForestPMPlot: A Flexible Tool for Visualizing Heterogeneity Between Studies in Meta-analysis. *G3 (Bethesda, Md.)* **6**, 1793-1798 (2016).
4. Lessard, C.J. *et al.* Variants at multiple loci implicated in both innate and adaptive immune responses are associated with Sjogren's syndrome. *Nat Genet* **45**, 1284-92 (2013).
5. Li, Y. *et al.* A genome-wide association study in Han Chinese identifies a susceptibility locus for primary Sjogren's syndrome at 7q11.23. *Nat Genet* **45**, 1361-5 (2013).
6. Pickrell, J.K. *et al.* Understanding mechanisms underlying human gene expression variation with RNA sequencing. *Nature* **464**, 768-772 (2010).
