# Supplementary material for: SLE non-coding genetic risk variant determines the epigenetic dysfunction of an immune cell specific enhancer that controls disease-critical microRNA expression
Source: Nat Commun. 2021 Jan 8;12:135. doi: 10.1038/s41467-020-20460-1 (PMC7794586; doi:10.1038/s41467-020-20460-1)
Supplement: Supplementary file 9 — Description of Additional Supplementary Files [file 41467_2020_20460_MOESM9_ESM.pdf]

Title: Supplementary Data 1:
Description: Discovery cohort and replication cohort study summary.

Title: Supplementary Data 2:
Description: DESeq2 results for all variants in MPRA experiment.

Title: Supplementary Data 3:
Description: Differential gene expression between WT group and KO group.

Title: Supplementary Data 4:
Description: Proteins binding with random and DNA sequence harboring rs2431697 identified by MS study.

Title: Supplementary Data 5:
Description: 4C-seq identified the interaction TSS regions with rs2431697 locus.

Title: Supplementary Data 6:
Description: miR-146a targets predicted by TargetScan, mirTarBase and miRDB.
