# Supplementary material for: SLE non-coding genetic risk variant determines the epigenetic dysfunction of an immune cell specific enhancer that controls disease-critical microRNA expression
Source: Nat Commun. 2021 Jan 8;12:135. doi: 10.1038/s41467-020-20460-1 (PMC7794586; doi:10.1038/s41467-020-20460-1)
Supplement: Supplementary file 10 — Reporting Summary [file 41467_2020_20460_MOESM10_ESM.pdf]

nature research

Corresponding author(s): Nan Shen

Last updated by author(s): Dec 3, 2020

Reporting Summary

Nature Research wishes to improve the reproducibility of the work that we publish. This form provides structure for consistency and transparency in reporting. For further information on Nature Research policies, see Authors & Referees and the Editorial Policy Checklist.

Statistics

For all statistical analyses, confirm that the following items are present in the figure legend, table legend, main text, or Methods section.

n/a	Confirmed	
☐	☒	The exact sample size (*n*) for each experimental group/condition, given as a discrete number and unit of measurement
☐	☒	A statement on whether measurements were taken from distinct samples or whether the same sample was measured repeatedly
☐	☒	The statistical test(s) used AND whether they are one- or two-sided<br>*Only common tests should be described solely by name; describe more complex techniques in the Methods section.*
☐	☒	A description of all covariates tested
☐	☒	A description of any assumptions or corrections, such as tests of normality and adjustment for multiple comparisons
☐	☒	A full description of the statistical parameters including central tendency (e.g. means) or other basic estimates (e.g. regression coefficient) AND variation (e.g. standard deviation) or associated estimates of uncertainty (e.g. confidence intervals)
☐	☒	For null hypothesis testing, the test statistic (e.g. *F*, *t*, *r*) with confidence intervals, effect sizes, degrees of freedom and *P* value noted<br>*Give P values as exact values whenever suitable.*
☐	☒	For Bayesian analysis, information on the choice of priors and Markov chain Monte Carlo settings
☒	☐	For hierarchical and complex designs, identification of the appropriate level for tests and full reporting of outcomes
☒	☐	Estimates of effect sizes (e.g. Cohen's *d*, Pearson's *r*), indicating how they were calculated

Our web collection on statistics for biologists contains articles on many of the points above.

Software and code

Policy information about availability of computer code

Data collection	BD FACSDIVA software (BD Biosciences, v8.0.1), QuantStudio Real-Time PCR software (ABI, v1.2), ImageJ 1.51, GAPipeline-1.6, HiSeq Control Software (HCS) (v3.3.76.1), OLB (v1.9.3).
Data analysis	GraphPad Prism 7 or 8, R version 3.3.3, ImageJ 1.51, PLINK v1.9, biomaRt_2.38.0, SNPTEST v 2.5.2, Picard (v2.19.0), Bowtie2(v2.3.5) or Bowtie-align (version 1.2) ,4Cseqpipe (version 0.7), circus (v0.69-6), MACS2(v2.1.2), Samtools (v0.1.19), cutadapt(v1.15), Hisat2 (v2.1.0), HT-seq (v0.11.2), DESeq2 (v1.24.0), Haploview (v4.1), Flowjo version 10, METASOFT (v2.0.1), ForestPMPlot (v1.0.3), METAL (v.2011-03-25), Eagle v2.4, Mascot Daemon (v2.3.0), Locuszoom (https://my.locuszoom.org and http://locuszoom.org, Formal versioning is not listed anywhere in the documentation on these websites).

For manuscripts utilizing custom algorithms or software that are central to the research but not yet described in published literature, software must be made available to editors/reviewers. We strongly encourage code deposition in a community repository (e.g. GitHub). See the Nature Research guidelines for submitting code & software for further information.

Data

Policy information about availability of data

All manuscripts must include a data availability statement. This statement should provide the following information, where applicable:

- Accession codes, unique identifiers, or web links for publicly available datasets
- A list of figures that have associated raw data
- A description of any restrictions on data availability

The RNA sequencing data, ATAC sequencing and 4C sequencing data that support the findings of this study have been deposited in ArrayExpress database under

accession codes E-MTAB-8978 (https://www.ebi.ac.uk/arrayexpress/experiments/E-MTAB-8978/), E-MTAB-8982 (https://www.ebi.ac.uk/arrayexpress/experiments/E-MTAB-8982/) and E-MTAB-9581(https://www.ebi.ac.uk/arrayexpress/experiments/E-MTAB-9581/) respectively. The mass spectrometry proteomics data that supports the findings have been deposited to the ProteomeXchange Consortium via the PRIDE partner repository with the dataset identifier PXD021335 (https://www.ebi.ac.uk/pride/archive/projects/PXD021335).

GWAS and Immunochip Summary statistics from three SLE association studies were downloaded from immunobase.org (http://urr.cat/data/GWAS_SLE_summaryStats.zip, now also available at https://www.ebi.ac.uk/gwas/studies/GCST005831) and (https://static-content.springer.com/esm/art%3A10.1038%2Fng.3496/MediaObjects/41588_2016_BFng3496_MOESM228_ESM.xlsx). The data from immunobase.org has since been re-located to https://www.ebi.ac.uk/gwas/studies/GCST003156. Genotype data for the eQTL analysis were accessed from the 1000 genomes project was downloaded from https://grch37.ensembl.org/Homo_sapiens/Tools/DataSlicer?db=core. Gene expression data for the eQTL analysis were downloaded from http://eqtl.uchicago.edu/RNA_Seq_data/results/ . All other remaining data are available within the Article and Supplementary Files or available from the authors upon request.

The source data underlying Figs.1a, 2a–d, 2f-g, 2i, 3a-f, 3h-j, 3l-n, 4d-e, 5b, 5d-f, 6a-d, 6f-g, Supplementary figs.1a–d, 2-8, 9a-c are provided as source data file.

Field-specific reporting

Please select the one below that is the best fit for your research. If you are not sure, read the appropriate sections before making your selection.

☒ Life sciences ☐ Behavioural & social sciences ☐ Ecological, evolutionary & environmental sciences

For a reference copy of the document with all sections, see nature.com/documents/nr-reporting-summary-flat.pdf

Life sciences study design

All studies must disclose on these points even when the disclosure is negative.

Sample size	We selected all DNA samples from SLE patients (as defined using the ACR classification criteria for lupus – Hochberg MC Arthritis Rheum 40:1725) and healthy control in Genetics in Discovery Cohort – LLAS2 study. A total of 17,003 DNA Samples used in this study and we retained 12, 733 individuals after quality control and potentially overlapping sample removal as detailed in the methods.<br><br>For Replication Cohort ,total 14,927 samples were used in Publicly available GWAS or Immunochip summary statistics from published papers: 1) Sun, C. et al. High-density genotyping of immune-related loci identifies new SLE risk variants in individuals with Asian ancestry. Nat Genet (2016). 2) Bentham, J. et al. Genetic association analyses implicate aberrant regulation of innate and adaptive immunity genes in the pathogenesis of systemic lupus erythematosus. Nat Genet 47, 1457-64 (2015). 3) Julia, A. et al. Genome-wide association study meta-analysis identifies five new loci for systemic lupus erythematosus. Arthritis Res Ther 20, 100 (2018).<br><br>For this study, in both the Discovery and Replication cohorts, no sample size calculations were performed. Sample size for the discovery cohort was determined in 2009 as a function of both available research funding and early experience with GWAS studies in SLE. [Nat Genet. 2008 Feb;40(2):204-10. doi: 10.1038/ng.81.] For the replication Cohort, the sample size was determined as a function of publicly available data with limited or known overlap with the Discovery cohort so that samples could be removed from the discovery cohort (as described in methods) to allow for independent statistical tests.<br><br>Other experiments were performed with two or more biological replicates as reported in the Statistics and figure legends. For animal study and human PBMCs study, the sample size is more than 5 to ensure robustness.
Data exclusions	For Genetics in Discovery Cohort, from a total of 17,003 individuals genotyped as part of this study, we retained 12, 733 individuals after quality control and potentially overlapping sample removal, this is described in method section. Similarly, samples from a potentially overlapping contributor, Dr. Sang Cheol-Bae were removed from the replication cohort. The purpose of these exclusions was to determine whether independent statistical evidence of genetic association was supported in this region AND to determine which variants were likely causal in the region.
Replication	All experiments were performed as biological replicates or biological samples replicates as appropriate for the experimental design.
Randomization	Animals were randomly allocated into either control or experimental group.
Blinding	Investigators were not blinded to group allocation as data analysis involved in all cases objective measurement methods are not affected by investigator bias.

Behavioural & social sciences study design

All studies must disclose on these points even when the disclosure is negative.

Study description	*Briefly describe the study type including whether data are quantitative, qualitative, or mixed-methods (e.g. qualitative cross-sectional, quantitative experimental, mixed-methods case study).*
Research sample	*State the research sample (e.g. Harvard university undergraduates, villagers in rural India) and provide relevant demographic information*

Research sample	(e.g. age, sex) and indicate whether the sample is representative. Provide a rationale for the study sample chosen. For studies involving existing datasets, please describe the dataset and source.
Sampling strategy	Describe the sampling procedure (e.g. random, snowball, stratified, convenience). Describe the statistical methods that were used to predetermine sample size OR if no sample-size calculation was performed, describe how sample sizes were chosen and provide a rationale for why these sample sizes are sufficient. For qualitative data, please indicate whether data saturation was considered, and what criteria were used to decide that no further sampling was needed.
Data collection	Provide details about the data collection procedure, including the instruments or devices used to record the data (e.g. pen and paper, computer, eye tracker, video or audio equipment) whether anyone was present besides the participant(s) and the researcher, and whether the researcher was blind to experimental condition and/or the study hypothesis during data collection.
Timing	Indicate the start and stop dates of data collection. If there is a gap between collection periods, state the dates for each sample cohort.
Data exclusions	If no data were excluded from the analyses, state so OR if data were excluded, provide the exact number of exclusions and the rationale behind them, indicating whether exclusion criteria were pre-established.
Non-participation	State how many participants dropped out/declined participation and the reason(s) given OR provide response rate OR state that no participants dropped out/declined participation.
Randomization	If participants were not allocated into experimental groups, state so OR describe how participants were allocated to groups, and if allocation was not random, describe how covariates were controlled.

Ecological, evolutionary & environmental sciences study design

All studies must disclose on these points even when the disclosure is negative.

Study description	Briefly describe the study. For quantitative data include treatment factors and interactions, design structure (e.g. factorial, nested, hierarchical), nature and number of experimental units and replicates.
Research sample	Describe the research sample (e.g. a group of tagged Passer domesticus, all Stenocereus thurberi within Organ Pipe Cactus National Monument), and provide a rationale for the sample choice. When relevant, describe the organism taxa, source, sex, age range and any manipulations. State what population the sample is meant to represent when applicable. For studies involving existing datasets, describe the data and its source.
Sampling strategy	Note the sampling procedure. Describe the statistical methods that were used to predetermine sample size OR if no sample-size calculation was performed, describe how sample sizes were chosen and provide a rationale for why these sample sizes are sufficient.
Data collection	Describe the data collection procedure, including who recorded the data and how.
Timing and spatial scale	Indicate the start and stop dates of data collection, noting the frequency and periodicity of sampling and providing a rationale for these choices. If there is a gap between collection periods, state the dates for each sample cohort. Specify the spatial scale from which the data are taken
Data exclusions	If no data were excluded from the analyses, state so OR if data were excluded, describe the exclusions and the rationale behind them, indicating whether exclusion criteria were pre-established.
Reproducibility	Describe the measures taken to verify the reproducibility of experimental findings. For each experiment, note whether any attempts to repeat the experiment failed OR state that all attempts to repeat the experiment were successful.
Randomization	Describe how samples/organisms/participants were allocated into groups. If allocation was not random, describe how covariates were controlled. If this is not relevant to your study, explain why.
Blinding	Describe the extent of blinding used during data acquisition and analysis. If blinding was not possible, describe why OR explain why blinding was not relevant to your study.

Did the study involve field work? ☐ Yes ☒ No

Field work, collection and transport

Field conditions	Describe the study conditions for field work, providing relevant parameters (e.g. temperature, rainfall).
Location	State the location of the sampling or experiment, providing relevant parameters (e.g. latitude and longitude, elevation, water depth).
Access and import/export	Describe the efforts you have made to access habitats and to collect and import/export your samples in a responsible manner and in compliance with local, national and international laws, noting any permits that were obtained (give the name of the issuing authority, the date of issue, and any identifying information).
Disturbance	Describe any disturbance caused by the study and how it was minimized.

Reporting for specific materials, systems and methods

We require information from authors about some types of materials, experimental systems and methods used in many studies. Here, indicate whether each material, system or method listed is relevant to your study. If you are not sure if a list item applies to your research, read the appropriate section before selecting a response.

Materials & experimental systems

n/a	Involved in the study
☐	☒ Antibodies
☐	☒ Eukaryotic cell lines
☒	☐ Palaeontology
☐	☒ Animals and other organisms
☐	☒ Human research participants
☒	☐ Clinical data

Methods

n/a	Involved in the study
☒	☐ ChIP-seq
☐	☒ Flow cytometry
☒	☐ MRI-based neuroimaging

Antibodies

Antibodies used

Anti-RELA (#8242S, Cell Signaling Technology, 1:100). Anti-H3K4me1 (#ab8895, Abcam, 2 µg for 25 µg of chromatin). Anti-H3K27ac (# ab177178, Abcam, 2 µg for 25 µg of chromatin). APC-H7 Mouse anti-Human CD45 (#560178, BD Biosciences, 1:50), BV421 Mouse Anti-Human CD19 (#562440, BD Biosciences,1:50), PE-Cy™7 Mouse Anti-Human CD14 (#562698, BD Biosciences,1:50), APC Mouse Anti-Human CD3 (#555335, BD Biosciences, 1:25).

Validation

Anti-RELA (#8242S, Cell Signaling Technology): Application: WB, IP, IHC, ChIP, ChIP-seq, IF, F, CUT&RUN. Species Reactivity: Human, Mouse, Rat, Hamster, Monkey, Dog.
Anti-H3K4me1 (#ab8895, Abcam): Application: ICC/IF, ChIP, WB, IHC-P. Species reactivity: Mouse, Human, Pig, Tetrahymena, Xenopus laevis, Plasmodium falciparum.
Anti-H3K27ac (#ab177178, Abcam): Application: Flow Cyt, ICC, PepArr, IHC-P, WB, ChIP, ChIP-seq. Species reactivity: Mouse, Rat, Human.
APC-H7 Mouse anti-Human CD45 (#560178, BD Biosciences): Application: Flow cytometry (Routinely Tested). Species Reactivity: Human (QC Testing).
BV421 Mouse Anti-Human CD19 (#562440, BD Biosciences): Application: Flow cytometry (Routinely Tested). Species Reactivity: Human (QC Testing).
PE-Cy™7 Mouse Anti-Human CD14 (#562698, BD Biosciences): Application: Flow cytometry (Routinely Tested). Species Reactivity: Human (QC Testing).
APC Mouse Anti-Human CD3 (#555335, BD Biosciences): Application: Flow cytometry (Routinely Tested). Species Reactivity: Human (QC Testing).

Eukaryotic cell lines

Policy information about cell lines

Cell line source(s)	U-937, Raji, Jurkat and HEK293T. All cell lines were obtained from Cell Bank of Chinese Academy of Science (Shanghai, China).
Authentication	All cell lines authenticated by STR method.
Mycoplasma contamination	All cell lines were tested negative for mycoplasma contamination.
Commonly misidentified lines (See ICLAC register)	No commonly misidentified cell lines were used.

Palaeontology

Specimen provenance	*Provide provenance information for specimens and describe permits that were obtained for the work (including the name of the issuing authority, the date of issue, and any identifying information).*
Specimen deposition	*Indicate where the specimens have been deposited to permit free access by other researchers.*
Dating methods	*If new dates are provided, describe how they were obtained (e.g. collection, storage, sample pretreatment and measurement), where they were obtained (i.e. lab name), the calibration program and the protocol for quality assurance OR state that no new dates are provided.*

☐ Tick this box to confirm that the raw and calibrated dates are available in the paper or in Supplementary Information.

Animals and other organisms

Policy information about studies involving animals; ARRIVE guidelines recommended for reporting animal research

Laboratory animals	6-8 weeks NOD-scid IL2Rγnull (NSG) female mice were used in our study and housed under a 12 hours light/12 hours dark cycle in a 23±2?temperature and 50±10% humidity.
Wild animals	No wild animals were used in this study.
Field-collected samples	No field-collected samples were used in this study
Ethics oversight	Animal experiments were approved by the Animal Care Committee of Renji Hospital, Shanghai Jiao Tong University School of Medicine (SJTUSM).

Note that full information on the approval of the study protocol must also be provided in the manuscript.

Human research participants

Policy information about studies involving human research participants

Population characteristics

Genetics: Discovery Cohort – LLAS2 – DNA Samples from SLE patients (as defined using the ACR classification criteria for lupus – Hochberg MC Arthritis Rheum 40:1725) and healthy control subjects were genotyped using the Illumina iSelect platform located at the Lupus Genetics Studies Unit at the OMRF. From a total of 17,003 individuals genotyped as part of this study, we retained 12, 733 individuals after quality control and potentially overlapping sample removal as detailed in the methods.

European & European American: 2337 cases, 1993 controls, 19.98% male. Asian & Asian American: 1257 cases 1258 controls, 9.74% male. Amerindian: 1475 cases, 805 controls, 9.07% male. African American: 1676 cases, 1932 controls, 20.02% male. Total: 6745 cases, 5988 controls.

Ancestral population group was self-reported and corroborated by principal component and admixture proportion calculations.

Replication Cohort – Publicly available GWAS or Immunochip summary statistics were used from published papers:
1) Sun, C. et al. High-density genotyping of immune-related loci identifies new SLE risk variants in individuals with Asian ancestry. Nat Genet (2016).
2) Bentham, J. et al. Genetic association analyses implicate aberrant regulation of innate and adaptive immunity genes in the pathogenesis of systemic lupus erythematosus. Nat Genet 47, 1457-64 (2015).
3) Julia, A. et al. Genome-wide association study meta-analysis identifies five new loci for systemic lupus erythematosus. Arthritis Res Ther 20, 100 (2018).
Population characteristics are described in those papers.
Briefly, 1) was a immunochip study of East Asian ancestry lupus patients and controls.
2) was a gwas study with imputation to the 1000 Genomes project of European ancestry lupus patients and controls and
3) was a gwas study with imputation to the 1000 Genomes project of European ancestry lupus patients and controls living in Spain.

eQTL analysis – Publicly available data from the 1000 Genomes project and RNA-Seq data from Pickrell, J.K. et al. Understanding mechanisms underlying human gene expression variation with RNA sequencing. Nature 464, 768-772 (2010) were reanalyzed to define eQTL relationship of rs2431697 and mir-146A expression as well as relationship of the locus to local and genome-wide eQTL traits across the whole genome. Population characteristics are described in that paper, but briefly, this is a group of African ancestry individuals of Yoruba ancestry living in Ibadan, Nigeria.

For CRISPR activation assay in PBMCs of SLE patients, SLE participants were 9 females (age of 40, 43, 47, 52, 41, 45, 18, 44, 52 years), and blood samples were collected for CRISPRi assay.

For PBMCs used in NSG humanized mice and in vitro editing, Health participants were 3 females and 3 males (age of 25, 26, 28, 32, 27, 26 years), and blood samples were collected for humanized mice construction and in vitro editing.

Recruitment

Discovery Cohort – Individuals were recruited by local investigators and informed consent was approved by the local institutional regulatory board.

Replication Cohort – Publicly available GWAS or Immunochip summary statistics were used as above in population characteristics.

eQTL analysis – as described in Pickrell, J.K. et al. Understanding mechanisms underlying human gene expression variation with RNA sequencing. Nature 464, 768-772 (2010), https://www.coriell.org/1/NHGRI/Collections/1000-Genomes-Collections/Yoruba-in-Ibadan-Nigeria-YRI, and Abecasis GR Nature 467(7319):1061

Further detailed description of recruiting efforts to the Lupus Family Registry and Repository, from which many of the discovery cohort samples were derived can be found at: https://pubmed.ncbi.nlm.nih.gov/20864496/. As for recruitment for the remainder of the discovery cohort, these samples were pooled part of an international fine-mapping and follow-up study (Large Lupus Association Study 2 – LLAS2) following initial GWAS efforts in SLE and as many samples as funding was available for were included for genotyping to maximize power to detect genetic association. Each individual was recruited from large lupus genetics studies centered in academic medical centers around the world (see below in oversight). Certainly, care or participation in studies centered in an academic medical center opens the possibilities of self-selection or referral bias based on disease severity.

If present such biases would tend to overestimate the impact of the genetic variants in this polygenic disease state.

For the human PBMCs used in CIRPSR activation assay, NSG humanized mice construction and in vitro editing, individuals were recruited by the Committee on Human Research of Renji Hospital, Shanghai Jiao Tong University School of Medicine (SJTUSM) and signed informed consent.

Ethics oversight

Oversight of the Discovery Cohort recruitment and consent process was overseen by the institutional review board of each contributing institution as described in: https://pubmed.ncbi.nlm.nih.gov/21194677/. Specifically, IRBs at Seattle Children's Hospital and University of Washington, Wake Forest University School of Medicine, University of California (Los Angeles), University of Southern California School of Medicine, University of Alabama at Birmingham, Medical University of South Carolina, Oklahoma Medical Research Foundation, CIB Rosario University, University of California (San Francisco) and Feinstein Institute of Medical Research, University of Oklahoma Health Sciences Center, University of Colorado School of Medicine, Hanyang University Hospital for Rheumatic Diseases,Mayo Clinic and King's College London provided recruitment oversight.

Oversight of the Replication Cohort and eQTL analyses was overseen by the institutional review board at the Cincinnati Children's Hospital Medical Center

For the human PBMCs used in CIRPSRi assay, NSG humanized mice construction and in vitro editing, All samples were collected with signed informed consent according to the the Committee on Human Research of Renji Hospital. The experiments were approved by the Committee on Human Research of Renji Hospital, Shanghai Jiao Tong University School of Medicine (SJTUSM).

Note that full information on the approval of the study protocol must also be provided in the manuscript.

Clinical data

Policy information about clinical studies

All manuscripts should comply with the ICMJE guidelines for publication of clinical research and a completed CONSORT checklist must be included with all submissions.

Clinical trial registration

Provide the trial registration number from ClinicalTrials.gov or an equivalent agency.

Study protocol

Note where the full trial protocol can be accessed OR if not available, explain why.

Data collection

Describe the settings and locales of data collection, noting the time periods of recruitment and data collection.

Outcomes

Describe how you pre-defined primary and secondary outcome measures and how you assessed these measures.

ChIP-seq

Data deposition

☐ Confirm that both raw and final processed data have been deposited in a public database such as GEO.

☐ Confirm that you have deposited or provided access to graph files (e.g. BED files) for the called peaks.

Data access links
May remain private before publication.

For "Initial submission" or "Revised version" documents, provide reviewer access links. For your "Final submission" document, provide a link to the deposited data.

Files in database submission

Provide a list of all files available in the database submission.

Genome browser session
(e.g. UCSC)

Provide a link to an anonymized genome browser session for "Initial submission" and "Revised version" documents only, to enable peer review. Write "no longer applicable" for "Final submission" documents.

Methodology

Replicates

Describe the experimental replicates, specifying number, type and replicate agreement.

Sequencing depth

Describe the sequencing depth for each experiment, providing the total number of reads, uniquely mapped reads, length of reads and whether they were paired- or single-end.

Antibodies

Describe the antibodies used for the ChIP-seq experiments; as applicable, provide supplier name, catalog number, clone name, and lot number.

Peak calling parameters

Specify the command line program and parameters used for read mapping and peak calling, including the ChIP, control and index files used.

Data quality

Describe the methods used to ensure data quality in full detail, including how many peaks are at FDR 5% and above 5-fold enrichment.

Software

Describe the software used to collect and analyze the ChIP-seq data. For custom code that has been deposited into a

Software	*community repository, provide accession details.*

Flow Cytometry

Plots

Confirm that:

☒ The axis labels state the marker and fluorochrome used (e.g. CD4-FITC).

☒ The axis scales are clearly visible. Include numbers along axes only for bottom left plot of group (a 'group' is an analysis of identical markers).

☐ All plots are contour plots with outliers or pseudocolor plots.

☒ A numerical value for number of cells or percentage (with statistics) is provided.

Methodology

Sample preparation	1×10^7 human PBMCs were injected into the NSG mice by tail vein injection. At 24 days post-inoculation, Mice were treated with 2×10^10 PFU adenovirus expressing Cas9-GFP and dual sgRNAs targeting rs2431697 region or negative control dual sgRNAs. After 3 days of transduction in vivo, the PBMCs and total spleen cells were isolated. The cells were stained directly with cocktails of fluorescently conjugated antibodies (APC-H7 Mouse anti-Human CD45 (560178, BD Biosciences), BV421 Mouse Anti-Human CD19 (562440, BD Biosciences), PE-Cy™7 Mouse Anti-Human CD14 (562698, BD Biosciences), APC Mouse Anti-Human CD3 (555335, BD Biosciences), 100 human CD45+CD3+GFP+ , 100 human CD45+CD19+GFP+ and 100 human CD45+CD14+GFP+ cells were sorted on a BD FACSAria III (Becton Dickinson).
Instrument	BD FACSAria III cell sorter
Software	software used to collect: BD FACSDiva Software Version 8.0.1<br>software used to analyze: FlowJo™ v10
Cell population abundance	Since the proportion of GFP+ human CD45+ cells is very low, we sorted the human CD45+CD3+ cells, human CD45+CD19+ and human CD45+CD14+ cells and analyzed the purity of these sorted cells by FACS, the collection tubes of post-sorted human CD45 +CD3+ cells, human CD45+CD19+ cells and human CD45+ CD14+ cells were reloaded to the flow cytometry respectively and detected under the sorting parameters. After sorting, the human CD45+ CD3+ cells purity was >96%, the human CD45+ CD19+ cells purity was >94% and the human CD45+ CD14+ cells purity was >89%.
Gating strategy	Using the FSC/SSC gating, debris was removed by gating on the main cell population. Then the human CD45+ cells were gated, and human CD3+ cells, human CD19+ cells and human CD14+ cells were separately gated from human CD45+ cells. Finally, the human CD3+GFP+ cells were gated from human CD45+CD3+ cells, human CD19+ GFP+cells were gated from human CD45+CD19 +cells, human CD14+ GFP+cells were gated from human CD45+CD14+cells.

☒ Tick this box to confirm that a figure exemplifying the gating strategy is provided in the Supplementary Information.

Magnetic resonance imaging

Experimental design

Design type	*Indicate task or resting state; event-related or block design.*
Design specifications	*Specify the number of blocks, trials or experimental units per session and/or subject, and specify the length of each trial or block (if trials are blocked) and interval between trials.*
Behavioral performance measures	*State number and/or type of variables recorded (e.g. correct button press, response time) and what statistics were used to establish that the subjects were performing the task as expected (e.g. mean, range, and/or standard deviation across subjects).*

Acquisition

Imaging type(s)	*Specify: functional, structural, diffusion, perfusion.*
Field strength	*Specify in Tesla*
Sequence & imaging parameters	*Specify the pulse sequence type (gradient echo, spin echo, etc.), imaging type (EPI, spiral, etc.), field of view, matrix size, slice thickness, orientation and TE/TR/flip angle.*
Area of acquisition	*State whether a whole brain scan was used OR define the area of acquisition, describing how the region was determined.*

Diffusion MRI ☐ Used ☐ Not used

Preprocessing

Preprocessing software	*Provide detail on software version and revision number and on specific parameters (model/functions, brain extraction, segmentation, smoothing kernel size, etc.).*
Normalization	*If data were normalized/standardized, describe the approach(es): specify linear or non-linear and define image types used for transformation OR indicate that data were not normalized and explain rationale for lack of normalization.*
Normalization template	*Describe the template used for normalization/transformation, specifying subject space or group standardized space (e.g. original Talairach, MNI305, ICBM152) OR indicate that the data were not normalized.*
Noise and artifact removal	*Describe your procedure(s) for artifact and structured noise removal, specifying motion parameters, tissue signals and physiological signals (heart rate, respiration).*
Volume censoring	*Define your software and/or method and criteria for volume censoring, and state the extent of such censoring.*

Statistical modeling & inference

Model type and settings	*Specify type (mass univariate, multivariate, RSA, predictive, etc.) and describe essential details of the model at the first and second levels (e.g. fixed, random or mixed effects; drift or auto-correlation).*
Effect(s) tested	*Define precise effect in terms of the task or stimulus conditions instead of psychological concepts and indicate whether ANOVA or factorial designs were used.*

Specify type of analysis: ☐ Whole brain ☐ ROI-based ☐ Both

Statistic type for inference (See Eklund et al. 2016)	*Specify voxel-wise or cluster-wise and report all relevant parameters for cluster-wise methods.*
Correction	*Describe the type of correction and how it is obtained for multiple comparisons (e.g. FWE, FDR, permutation or Monte Carlo).*

Models & analysis

n/a	Involved in the study
☐	☐ Functional and/or effective connectivity
☐	☐ Graph analysis
☐	☐ Multivariate modeling or predictive analysis

Functional and/or effective connectivity	*Report the measures of dependence used and the model details (e.g. Pearson correlation, partial correlation, mutual information).*
Graph analysis	*Report the dependent variable and connectivity measure, specifying weighted graph or binarized graph, subject- or group-level, and the global and/or node summaries used (e.g. clustering coefficient, efficiency, etc.).*
Multivariate modeling and predictive analysis	*Specify independent variables, features extraction and dimension reduction, model, training and evaluation metrics.*
